# Insights into the Sex-Related Effects of Dietary Polyphenols and Metabolic Disruptors on Inflammatory and (Neuro) Endocrine Pathways in Obesity: The HEAL Project

**DOI:** 10.3390/nu16213595

**Published:** 2024-10-23

**Authors:** Carmela Santangelo, Beatrice Scazzocchio, Rosaria Varì, Maria Antonietta Ajmone-Cat, Alessia Tammaro, Sabrina Tait, Irene Masciola, Roberta Tassinari, Olimpia Vincentini, Rita Di Benedetto, Alessandra Berry, Francesca Cirulli, Francesca Maranghi, Roberta De Simone, Massimo D’Archivio

**Affiliations:** 1 Gender-Specific Prevention and Health Unit, Centre for Gender-Specific Medicine, Istituto Superiore di Sanità, 00161 Rome, Italy; carmela.santangelo@iss.it (C.S.); rosaria.vari@iss.it (R.V.); alessia.tammaro@guest.iss.it (A.T.); sabrina.tait@iss.it (S.T.); irene.masciola@guest.iss.it (I.M.); roberta.tassinari@iss.it (R.T.); francesca.maranghi@iss.it (F.M.); massimo.darchivio@iss.it (M.D.); 2 National Center for Drug Research and Evaluation, Istituto Superiore di Sanità, 00161 Rome, Italy; mariaantonietta.ajmone-cat@iss.it; 3 Department of Biomedicine and Prevention, Tor Vergata University of Rome, 00133 Rome, Italy; 4 Human Nutrition and Health Unit, Department of Food Safety, Nutrition, and Veterinary Public Health, Istituto Superiore di Sanità, 00161 Rome, Italy; olimpia.vincentini@iss.it (O.V.); rita.dibenedetto@iss.it (R.D.B.); 5 Center for Behavioral Sciences and Mental Health, Istituto Superiore di Sanità, 00161 Rome, Italy; alessandra.berry@iss.it (A.B.); francesca.cirulli@iss.it (F.C.)

**Keywords:** obesity, polyphenols, metabolic disruptors, nutrition, diet, metabolic disorders, neuro-inflammation

## Abstract

Background: this study was performed under the umbrella of the Health Extended Alliance for Innovative Therapies, Advanced Lab Research, and Integrated Approaches of Precision Medicine (HEAL ITALIA) partnership and funded under the National Recovery and Resilience Plan, Mission 4 Component 2 Investment 1.3, and by the European Union. Objectives: the overall objective of the HEAL project is to identify innovative and effective therapeutic approaches to reduce disease burden. The present research falls within Spoke 7: Prevention Strategies: Integrated and gender medicine approaches for prevention strategies based on environmental, lifestyle, and clinical biometric data. Obesity represents a primary risk factor worldwide for the onset of numerous life-threatening diseases, including metabolic, cardiovascular, and neurological diseases. Environmental and gender-related factors influence obesity development. However, the molecular mechanisms underlying the effects of those agents on different organs of the human body are not fully understood yet. Methods: here, we present a study protocol aimed at shedding light on (i) the complex interplays among adipose tissue, brain and gut in obesity, and (ii) the effects of specific dietary components and environmental metabolism-disrupting compounds on those interactions. To this purpose, we combined ex vivo, in vitro, and in vivo approaches to gain additional knowledge about the molecular mechanisms underlying connections between organs. Conclusions: the data provided by this study will contribute to defining new targets for therapeutic and/or preventive interventions, thereby allowing more personalized approaches to nutrition.

## 1. Introduction

This study was carried out as part of the partnership between Health Extended Alliance for Innovative Therapies, Advanced Lab-research, and Integrated Approaches of Precision Medicine (HEAL ITALIA). It was funded under the National Recovery and Resilience Plan (NRRP), Mission 4 Component 2 Investment 1.3, and by the European Union—NextGenerationEU to increase partnerships between universities and clinical and research institutes.

The HEAL ITALIA partnership comprises a multidisciplinary network of laboratories, clinical research centers, and enterprises, sharing knowledge and technologies to reach results with timeliness, increase the quality of health services, and ultimately carry the Italian National Health System into the contemporary era of precision medicine.

The overall objective of the HEAL project is to deliver new, cost-effective, and evidence-based predictive and non-invasive diagnostic pathways for faster, earlier, and affordable prediction, detection, and monitoring of monogenic (rare diseases) and polygenic (cardiovascular and metabolic) disorders and cancer. It also aims to identify innovative and effective therapeutic approaches. The HEAL project consists of eight distinct spokes divided into highly interrelated Work Packages and Tasks. The present project falls within the scope of Spoke 7 (Prevention Strategies: Integrated and gender medicine approaches for prevention strategies based on environmental, lifestyle, and clinical biometric data); WP 3 (Integrating old risk factors and novel predictive models for the prevention of Metabolic and Endocrine Diseases); Task 3.1: Risk factors, lifestyle, and new biomarkers in obesity and related diseases.

Obesity is recognized as an epidemic worldwide as the rates of being overweight and obese continue to grow in adults and children (https://www.who.int/news-room/fact-sheets/detail/obesity-and-overweight (accessed on 10 October 2024)), especially in many low and middle-income countries [1]. The worldwide prevalence of obesity has more than doubled between 1990 and 2022 (https://www.who.int/news-room/fact-sheets/detail/obesity-and-overweight (accessed on 10 October 2024)). Obesity represents a costly condition since it leads to disability and decreased productivity. Moreover, an even more serious concern is that obesity represents a primary risk factor for the onset of numerous life-threatening diseases, including metabolic, cardiovascular, and neurological diseases, which can reduce life expectancy [2]. Despite this awareness, attempts to prevent and manage obesity and its consequences are, so far, ineffective and unsuccessful. Several factors contribute to obesity, which represents a multifactorial disease reflecting a multifaceted interplay of genetic, epigenetic, and obesogenic environmental factors [3,4,5].

Unhealthy dietary habits and reduced physical activity are considered the main modifiable environmental factors [6]. Recent studies have indicated different nutritional habits and health beliefs between men and women that may influence their dietary behavior and food choices. Women tend to choose healthier food while men prefer animal-derived food [7]. To date, unhealthy nutrition based on red meat, processed food, and sugar-, salt- and fat-rich food and drink consumption may induce and/or exacerbate the inflammatory process, thereby promoting the development of non-communicable diseases (NCDs) [8]. Moreover, recent data show that sex/gender differences in obesity prevalence as well as body fat distribution and accumulation exist [9], resulting in different obesity-related alterations [10]. Very few dietary intervention studies have been designed that consider sex/gender differences, which is actually fundamental to managing human diseases.

It is well known that the Mediterranean diet, based on fruits, vegetables, legumes, nuts, grains, as well as spices, herbs, and seeds, is nutritionally rich and high in polyphenols, which have anti-inflammatory and antioxidant activities and exert protective effects on human health [11,12,13]. Among dietary polyphenols, anthocyanins are abundant in colored fruits, vegetables, and whole grains. They can modulate various mechanisms linking obesity, chronic inflammation, and gut dysfunction [14] with insulin resistance in type 2 diabetes (T2D) [15] and neurodegenerative diseases [16]. Our group and others have shown that protocatechuic acid (PCA), one of the main metabolites of anthocyanins [17], exerts insulin-like activity, and counteracts the inflammatory milieu by modulating several signaling pathways in human adipocytes [18,19,20] and rat models of insulin resistance [18]. In addition, in vitro and in vivo studies have shown that PCA’s antioxidant and anti-inflammatory properties make it a therapeutic candidate for treating neurodegenerative diseases [15]. Finally, a large number of recent studies have found that PCA is associated with countless activities including antioxidant, neuroprotective, and anti-inflammatory effects as well as antibacterial, antiviral, anti-cancer, anti-osteoporotic, analgesic, anti-aging, and reproductive functions. Future preclinical and clinical research should explore the pharmacological activities of PCA in light of these findings [21].

In addition to unhealthy dietary habits, some compounds previously classified as endocrine disruptors, now recognized as metabolism-disrupting chemicals (MDCs), can be considered obesogenic environmental factors. In fact, they can alter metabolic set-point control [22,23], damage the central nervous system [24], and alter gut functions [25].

Several widely used pesticides to protect agricultural crops from pest-related damage and kill other pests are recognized as MDCs [26]. The accumulation of pesticides in adipose tissue (AT ) suggests that, by altering the function of adipocytes, they contribute to adiposity development [27]. MDCs appear to affect the development and function of AT differently in men and women [28]. Some evidence indicates that the group of neonicotinoids may represent risk factors for obesity onset; for example, acetamiprid (ACE), a widely used neonicotinoid, is extremely toxic to insects but has a high affinity for mammalian nicotinic acetylcholine receptors. Exposure to ACE provokes organ system toxicity by disrupting immune physiology and ion balance [29]. An epidemiological study from the United States indicated that detectable concentrations of distinct MDCs, including ACE, have different associations withmeasures of obesity in adults and are gender-specific [30]. ACE stimulated in vitro adipocyte differentiation [31], whereas ACE treatment increased triglyceride levels in male mice [32].

Although growing evidence indicates that natural compounds found in fruits and vegetables can help mitigate the toxic effects of pesticides [24], further research is needed to clarify the mechanisms and dosages by which these compounds protect against pesticide effects.

Over the past few decades, a connection between the AT, central nervous system, and gut has been unveiled [33,34]. Obesity has been associated with an increased risk of cognitive decline [35] and gut dysbiosis [36]. The AT secretes a multiplicity of adipokines, lipokines, and pro- and anti-inflammatory cytokines that reach the brain through peripheral circulation [37,38]. In obese people, the expression of pro-inflammatory factors increases, affecting several organs including the brain, causing neuroinflammation and dysfunction in brain regions [37,38].

The exact mechanisms of AT/brain communication are complex and not fully understood. However, they involve the activation of microglia, the resident immune cells of the brain, in a pro-inflammatory state [37,38]. Among the involved brain areas, the hypothalamus is the main regulator of different body axes, including the hypothalamic–pituitary–gonadal (HPG) axis. It is also a sensitive target of pesticide exposure [39,40]. Increased hypothalamic inflammation may lead to early-onset puberty due to the precocious activation of gonadotropin-releasing hormone (GnRH) neurons [41]. Overall, the crosstalk between AT and the brain in obesity can affect the homeostasis of neural cells, leading to an increased risk of brain dysfunction and pathologies as well as metabolic disorders and their consequences, including reproductive disorders, with sex-specific outcomes.

The primary aim of this study was to compare specific inflammatory factors/pathways in obese men and women. We hypothesized that (i) a different expression of those factors both in AT and blood and (ii) a different response of AT to ACE and PCA treatment would be observed according to sex.

The secondary aim was to shed light on the molecular mechanisms by which inflammatory factors released by AT from obese men and women modulate the response of brain and intestinal cell lines differently to ACE and PCA treatment.

The third aim was to evaluate the potential involvement of ACE—associated or not associated with high-fat diet consumption—in the onset of metabolic and neurological diseases as well as the protective effects of PCA in male and female juvenile rats.

The strength of this project lies in combining different approaches, namely ex vivo, in vivo, and in vitro studies, focusing on sex/gender differences. The knowledge gained from this study will contribute to defining new targets for preventive and therapeutic interventions that can restore normal tissue functions and provide data promoting science-based measures. The aim of this research was consistent with the expected impact of Spoke 7 of the HEAL Project: “to improve disease prevention, thus reducing morbidity and mortality and the resulting economic burden on the National Health System”.

### Study Objectives

Ex vivo: To evaluate the effects of a specific nutrient (PCA) and MDCs (ACE) on AT obtained from obese men and women undergoing bariatric surgery. The underlying molecular mechanisms were analyzed taking into account sex, age, health status, and dietary intake. The dietary intake of each obese subject was associated with inflammatory status by evaluating the expression of specific factors in peripheral blood.

In vitro: To assess the interactions between AT, the gut, and the brain by treating human microglial cell lines, mouse hypothalamic GnRH expressing neurons, and human intestinal cell lines, with conditioned media from ATs (ATCM) derived from obese men and women undergoing bariatric surgery. The cells were exposed or not exposed to PCA, ACE, or their combination, and specific inflammatory and neuroendocrine pathways were analyzed.

In vivo: To assess the contribution of ACE to the onset of metabolic and neurological diseases and the potential protective effects of PCA in juvenile rodents of both sexes exposed to a standard or obesogenic Western diet. To this end, by considering sex and age-related vulnerability and susceptibility to treatments, we evaluated the expression of specific biomarkers in AT, the gut, and the brain that may be transferable to human studies.

## 2. Methodology

### 2.1. Design

This study will integrate different approaches: (i) ex vivo model using visceral AT biopsies derived from 80 obese subjects (40 men and 40 women); (ii) in vitro models using human microglial, murine hypothalamic, and human intestinal cell lines; (iii) in vivo model using juvenile male and female rats.

### 2.2. Human Subjects

The subjects will be recruited by the Complex Operational Unit of General and Hepatobiliary Surgery of St. Andrea University Hospital and evaluated approximately two weeks before surgery. During the first visit, a general clinical examination will be performed, and 10 mL of fasting blood will be taken for standard measurements of hematological and clinical parameters. Waist circumference, height, and weight will also be determined to calculate the body mass index (BMI). Visceral adipose tissue (AT) biopsies will be collected during surgery as specified in paragraphs 2.7 and 2.8. Subjects will participate on a voluntary basis. Interested subjects will be provided with information on the study and asked to sign informed consent to the participation and collection of biological samples for carrying out the research project. The biological samples and questionnaires will be in pseudonymized form, i.e., with the attribution of a unique identification code without the possibility of identifying the participants in the study. Only authorized personnel involved in carrying out this research will be able to link this code to the name of the participant.

### 2.3. Eligibility Criteria

Obese subjects undergoing surgery for bariatric surgery (age 21–75; BMI > 30) will be considered eligible for the study.

### 2.4. Exclusion Criteria

Subjects will be excluded if they tested positive for COVID-19 in the last 6 weeks, have clinical evidence of active infection, chronic diabetes; recently used antibiotics or anti-inflammatory drugs (within 14 days); underwent radiotherapy, chemotherapy, or steroid or cortisone anti-inflammatory therapies; abused drugs or alcohol, experienced chronic renal failure, have neoplastic pathologies, are pregnant, have a mental disability, or a diagnosed eating disorder.

### 2.5. Ethical Considerations

The investigation will be conducted in accordance with ethical standards, the Declaration of Helsinki, and national and international guidelines. This study has been approved by the National Ethics Committee (Istituto Superiore di Sanità AOO-ISS-03/05/2023-0020590 Class: PRE BIO CE 01.00). All enrolled subjects will be provided with complete information about the study and asked to sign informed consent.

### 2.6. Nutritional Questionnaires

To estimate the usual food intake and physical activity level, validated questionnaires [42] will be self-administered to the participants. The questionnaires will be collected and stored in a special locked cabinet at St. Andrea University Hospital in a specific locked room. They will be sent monthly to the Istituto Superiore di Sanità (ISS) via a specialized courier. Energy and nutrient intake will be calculated using WINFOOD software (Version n. 3.9) based on the European Institute of Oncology and INRAN Food Composition Tables (2000). Based on the answers obtained from the physical activity questionnaire, the subjects will be classified into three categories (low, moderate, and vigorous activity).

### 2.7. Transport of Human Biological Samples

The biological samples will be stored in a physiological solution and immediately shipped to ISS via a specialized courier, guaranteeing the correct preservation of the ATs. During transport, all safety conditions for the operator and the samples will be implemented, preventing the dispersion of potentially infectious agents into the environment. The entire cycle of operations, starting from packaging and transport up to the reception of the materials, must guarantee the safety of all workers (laboratory personnel and transport service workers) and the environment.

### 2.8. Adipose Tissue Culture

AT biopsies will be rinsed several times in 0.9% NaCl, then cut into pieces weighing 200 mg and cultured in low-glucose Dulbecco’s Modified Eagle’s Medium (DMEM) (1000 mg/L D-(+)-glucose) with or without PCA 100 µM [19]. This concentration has been already demonstrated not to be toxic and can exert several functions on human AT [19]. To define the lowest effective concentration of ACE capable of modulating AT activities, we will carry out preliminary experiments, incubating ATs with different concentrations of ACE (10–150 nM) at different times (24–48 h). Once the optimal experimental conditions are defined, treated ATs and corresponding AT-conditioned media (ATCM) will be collected and stored at −80 °C for subsequent specific analyses. The following four groups: untreated AT (control), AT with PCA, AT with ACE, and AT with PCA + ACE, will be analyzed.

### 2.9. Human ATCM Effects on Different Cell Types

Human microglia cell line: We will use the human microglial cell line clone 3 (HMC3; ATCC^®^CRL-3304), a widely studied and extensively characterized experimental model with respect to cell morphology, antigenic profile, and cell functions [43]. These cells retain the typical antigenic profile and functional properties of microglia and can respond to a pattern of chemokines and inflammatory stimuli. HMC3 will be expanded in low-glucose DMEM, 10% fetal calf serum (FCS), 2 mM glutamine, and antibiotics, sub-cultured, and stored in frozen aliquots at passage 3 to have a homogeneous starting population for the experiments of the entire project. Cells will be used between passages 3 and 10, and all experiments will be performed in serum-free, low-glucose DMEM. We will also evaluate possible direct immunomodulatory and/or toxic effects of different doses of ACE, PCA, and their combination on sister HMC3 microglial cultures, with or without the presence of typical inflammatory stimuli, such as lipopolysaccharide (LPS). The optimal dose of 100 ng/mL LPS, which elicits a typical microglial inflammatory response, will be used. To evaluate possible alterations in the crosstalk between AT and microglia, the brain resident immune cells, HMC3 cells will be exposed at different times, ranging from 12 to 96 h, to ATCMs from treated ATs. At the end of the treatments, the microglial supernatants and cell pellets will be harvested for molecular and biochemical analyses of gene and protein expression, or the cultures will be subjected to functional assays, such as phagocytosis assays, as previously described [44].

Mouse hypothalamic immortalized GnRH neurons: The mouse hypothalamic cell line of immortalized GnRH neurons (GT1-7), kindly provided by Prof. Pamela Mellon [45], will be cultured in DMEM without phenol red (Gibco, Milan, Italy), supplemented with 10% fetal bovine serum (FBS), 100 U/mL penicillin, 100 µg/mL streptomycin, and 2 mM L-Glutamine. Cells will be maintained in an incubator at 37 °C, 5% CO_2_, and 90% humidity. GT1-7 cells will be exposed to PCA and ACE, alone or in combination, to assess their direct cytotoxicity to these cells. Then, cells will be treated for 72 h with ATCMs. Supernatants and cell pellets will be harvested for gene and protein expression analysis of a panel of inflammatory and neuroendocrine biomarkers. In addition, immunofluorescence staining of neuronal markers will be performed.

Human intestinal cell line: The in vitro intestinal model of Caco-2 cells (HT-B 37 clone- human colorectal adenocarcinoma) will be used to simulate the intestinal barrier. Caco-2 cells will be obtained from the European Collection of Cell Culture (ECACC, Porton Down, Salisbury, UK). Cells will be cultured in high glucose DMEM supplemented with 10% FBS, 1% non-essential amino acids, 1% L-glutamine, and 1% penicillin and streptomycin (PEST). Caco-2 cells will be sub-cultured at 90% confluence and seeded, as previously described [46]. These cells, although of colonic origin, can spontaneously differentiate in long-term culture to an enterocytic phenotype. The cells will be cultured on a transwell system (1 μm pore size insert) to allow polarization, where brush border enzyme secreting microvilli develop on the apical side, and uniform tight junctions form between adjacent cells. At the end of the differentiation process (day 21), the cells will be exposed for 24 h from the apical part to PCA alone and in combination with ACE. Barrier integrity will be monitored by measuring both transepithelial resistance (TEER) and permeability by Lucifer Yellow translocation. To unravel inflammatory signaling between intestinal epithelial cells and adipocytes, Caco-2 cells will also be exposed in the basolateral part to ATCM to evaluate possible networks with adipocytes exposed to PCA and ACE. Supernatants and cell pellets will be harvested for gene and protein expression analysis and cytokine release and barrier integrity will be monitored.

### 2.10. Animal Studies

All animal studies will be executed in accordance with Directive 2010/63/EU, the Italian Legislative Decree n. 26 of 4 March 2014, the OECD Principles of Good Laboratory Practice. The study protocol has been approved by the Italian Ministry of Health (737/2023_PR). The design and sample size of the animal studies will be chosen according to 3R principles and ARRIVE guidelines 2.0 (https://arriveguidelines.org (accessed on 10 October 2024)).

The first study aims to identify the lowest dose level of ACE capable of inducing metabolic alterations in 64 juvenile Sprague–Dawley rats of both sexes (32 females and 32 males—approx. body weight 40–50 g), housed two per cage according to sex and treatment group, during the peripubertal period from weaning to full sexual maturity—corresponding to the childhood phase in humans. The dose levels of 0, 0.07, 0.7, and 7 mg/kg bw per day are derived from the systemic NOAEL of 7.1 mg/kg based on body weight reduction in females and histopathological effects on the liver of male rats in the 2-year study [47]. ACE will be administered by gavage for 28 days (5 days/week).

The second study will evaluate the effects and mode of action of ACE and PCA in target tissues (AT, liver) and brain-gut axis of 216 juvenile Sprague–Dawley rats of both sexes (108 females and 108 males–approx. body weight 40–50 g), housed two per cage according to sex and treatment group, fed with a standard or high-fat rodent diet. The dose level of ACE will be selected from the first study. The dose of 100 mg/kg PCA is reported in literature data concerning PCA’s potential protective effects on metabolic diseases [20]. ACE and PCA in a mixture will be administered by gavage for 28 days (5 days/week). Body weight, feed consumption, organs (brain, liver, AT, and gut) as well as absolute and relative weights will be measured. Serum clinical and biochemical parameters (albumin, globulin, total bilirubin, gamma-glutamyl transferase, aspartate aminotransferase, alkaline phosphatase, lactate dehydrogenase, creatine kinase, creatinine, glucose, triglycerides, calcium, phosphate, magnesium, potassium, and sodium), histopathological analysis and gene and protein expression of selected inflammatory and oxidative stress markers in the brain, liver, AT, and gut will be evaluated using q-PCR and Western blot (WB) techniques, as described below.

### 2.11. Inflammatory and Oxidative Marker Analysis by ELISA Assays

Plasma, AT-conditioned media (ATCM), Caco-2, and microglial supernatants will be analyzed for specific inflammatory parameters (IL-6, TNFα, IL10, leptin, and adiponectin), oxidative stress markers such as ROS, oxLDL, Catalase and Copper-zinc superoxide dismutase (Cu-Zn SOD) using ELISA assays. GnRH release, IL-10, and TNFα levels will be evaluated in GT1-7 supernatants by commercial ELISA assays.

### 2.12. Protein Determination by Western Blot Analysis

After treatment, AT samples, intestinal, microglial, and hypothalamic cell pellets as well as brain, liver, AT, and gut rat samples will be lysed in a lysis buffer to obtain tissue extracts, as previously described [19,40,48]. Immunoblotting analyses will be carried out using specific antibodies directed against nuclear receptors, transcription factors, and kinases involved in the modulation of hormonal, metabolic and inflammatory pathways, such as PPARγ, PPARα, p-NFkB, NFkB, ERα, ERβ, AR, CYP19A1, GSK-3β, p66shc, and against enzymes involved in oxidative stress pathways such as MnSOD and catalase, or neuronal functional markers such as Map2. Blots will be treated with appropriate secondary antibodies conjugated with horseradish peroxidase followed by ECL detection. Equal loading of proteins will be verified by immunoblotting with anti-β-actin or GAPDH antibodies. Densitometric analysis will be performed using a molecular imager FX (Bio-Rad, Hercules, CA, USA).

### 2.13. RNA Extraction, Reverse Transcription, and Real-Time PCR Analyses

For transcript analyses of pro- and anti-inflammatory markers, oxidative stress-related enzymes, neuroendocrine markers, and genes involved in phagocytosis, RNA will be extracted from AT samples, intestinal, microglial, and hypothalamic cell pellets as well as brain, liver, AT, and gut rat samples using the Norgen RNA kit (Norgen, Thorold, ON, Canada). A total of 1 µg of RNA from each sample will be reverse transcribed to cDNA by the SensiFast™ cDNA Synthesis Kit (Bioline Reagents Ltd., London, UK). The Excel TaqTM Fast Q-PCR Master Mix SYBR (SMOBIO Technology Inc., Hsinchu City, Taiwan) will be used to prepare PCR reactions, run on a Bioer LineGene 9600 Plus thermocycler instrument (Bioer Technology Co., Ltd., Hangzhou, China). Threshold cycles (Ct) obtained by the LineGene 9600 PCR V.1.0 software (Bioer) will be used to calculate ∆Ct values with control cells as calibrators and Gapdh, HPRT, or b-actin as normalizers.

### 2.14. Statistical Methods

Continuous data will be presented as the mean and standard deviation or, when appropriate, in terms of the median and 25th–75th percentile. Categorical data will be presented in terms of count and percentage. A *p*-value < 0.05 will be considered statistically significant.

For continuous variables, the measure of central tendency (mean and median) and dispersion (standard deviation and percentiles) will be calculated. After having assessed whether the distribution is normal by the Shapiro–Wilk test, inferential statistics will be performed with parametric (student’s *t*-test; ANOVA) or non-parametric (Kruskal–Wallis) tests to identify significant differences between groups. Furthermore, post-hoc tests will be performed, whenever applicable, for pairwise comparisons (Dunnet or Dunn, according to the distribution). For qualitative variables, frequencies and confidence intervals will be calculated. The two-tailed chi-square test will be used to evaluate statistical differences between groups.

Correlations between quantitative variables will be performed using parametric or non-parametric tests depending on the distribution. In order to analyze the correlation between quantitative variables (e.g., levels of inflammatory biomarkers) and qualitative data (i.e., data from nutritional questionnaires), “fictitious” (dichotomous) qualitative variables will be created according to the percentiles (over 50%, over 75%, and over 90%).

A data quality check will also be performed on the completed questionnaires to identify any biases due to data collection.

## 3. Expected Results

The results of this study will highlight possible differences in the expression of specific inflammatory pathways in obese individuals according to sex/gender and dietary habits. We expect to gain a deeper understanding of the activation of different molecular mechanisms in men and women following the treatment of AT with MDCs and dietary components.

Moreover, the project will elucidate the cellular interactions between AT and the brain, as well as between AT and the gut. We assume that the AT of men and women will have different effects on the inflammatory state of intestinal cells, microglial homeostasis and reactivity, and hypothalamic signaling.

Finally, the animal studies will enhance our understanding of the mechanisms associated with the effects of ACE and PCA on the endocrine–metabolic system, AT, and the gut–brain axis during the susceptible and vulnerable peripubertal phase of life.

The insights gained from this study hold potential for identifying novel therapeutic and/or preventive targets, including dietary interventions, aimed at restoring normal tissue functions, thus improving personalized nutrition, which considers the different metabolic and hormonal needs of men and women.
